# An Unusual Case of Stercoral Perforation in a Patient with 86 cm of Small Bowel

**DOI:** 10.1155/2013/317250

**Published:** 2013-09-09

**Authors:** Alfin Okullo, Ghiyath Alsnih, Titus Kwok

**Affiliations:** ^1^Department of Surgery, Blacktown-Mt Druitt Hospital, Blacktown Road, Blacktown, Sydney, NSW 2148, Australia; ^2^Department of Surgery, Concord Repatriation and General Hospital, Hospital Road, Concord, Sydney, NSW 2139, Australia

## Abstract

A 77-year-old male who previously had extensive enterectomy due to ischaemic gut with loss of all but 86 cm of jejunum in addition to a right hemicolectomy presented to the emergency department (ED) with abdominal pain and constipation of 12-day duration. Abdominal imaging with X-ray and CT revealed pneumoperitoneum in addition to a grossly redundant and faecally loaded colon. At laparotomy, rectal perforation was found. In view of the patient's advanced age, comorbidities, and the absence of intraperitoneal faecal contamination, manual disimpaction followed by wedge resection and primary closure of the perforation was done. On postop day 11, a perforation in the sigmoid colon with free subdiaphragmatic gas was picked up on CT after a work up for abdominal tenderness. In the absence of peritonism and other signs of deterioration, conservative management was chosen with subsequent uneventful recovery for the patient.

## 1. Introduction

A patient with 86 cm of small bowel is expected to have short bowel syndrome (SBS). It is quite unusual for such a patient to present with constipation so severe that it causes stercoral perforation. We present a rare case of one such patient.

## 2. Case Report

A 77-year-old male presented to the (ED) with generalized abdominal pain and constipation of about 12-day duration. He was still passing wind and denied any nausea or vomiting.

His previous surgical history consisted of an extensive enterectomy 17 years ago for ischaemic gut, whereupon all but 86 cm of his jejunum was resected in addition to a right hemicolectomy. A jejunal-transverse colon anastomosis was done. Back then, his postop recovery was notable for a quick resolution of his diarrhea while on a low-fibre diet without any antimotility drugs and a return to his premorbid, chronically constipated state within about 2 months of the surgery. He thereafter required daily lactulose whose dose he had increased from 20 to 30 mls to no avail over the past 12 days. He was never on any medicines used to treat short bowel syndrome. A review of his regular medications was negative for any that would contribute to his chronic constipation prior to and after the bowel resection 17 years ago.

 At this presentation, his vital signs and blood tests were all normal except for an INR of 6.6.

**Figure 1:**
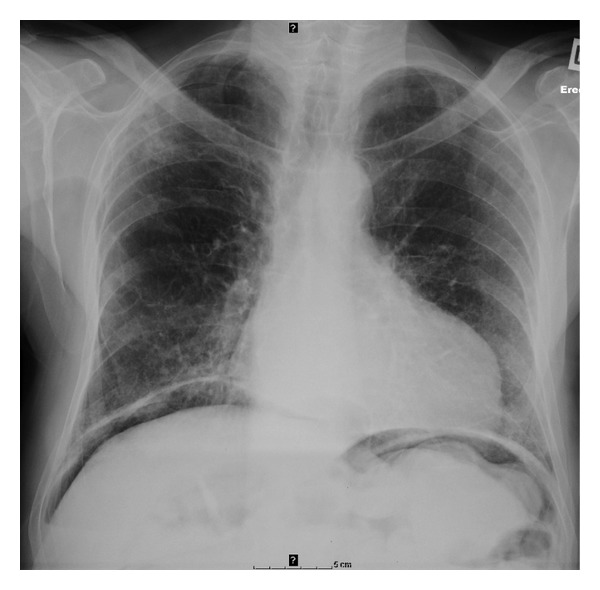
CXR demonstrating air under the diaphragm on presentation.

 A chest X-ray revealed free subdiaphragmatic gas (Figure 1), while a plain erect abdominal X-ray showed gross faecal loading.

**Figure 2:**
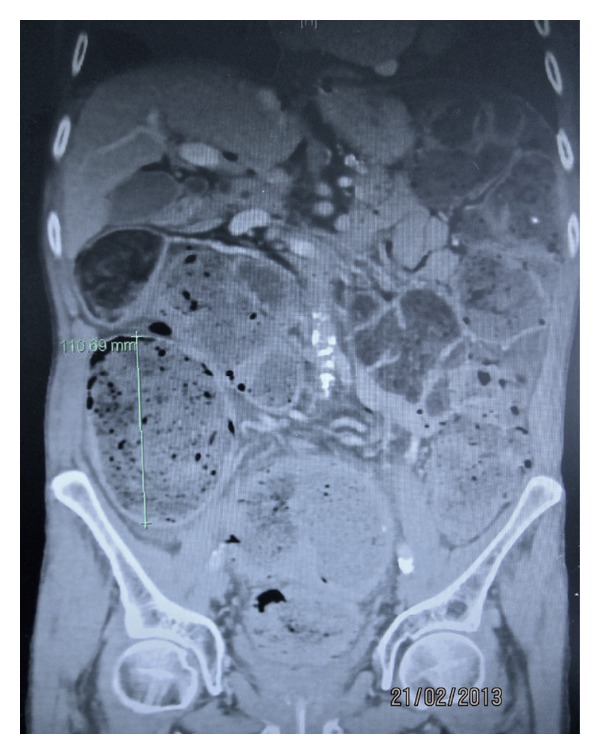
CT abdomen and pelvis demonstrating gross faecal loading and colonic dilatation.

A CT abdomen and pelvis with oral and intravenous contrast demonstrated gross faecal loading in the colon up to the rectum with a very large amount of free intraabdominal gas. There was a moderate amount of gas present within a grossly redundant sigmoid colon with colonic diameter of up to 110 mm in some sections (Figure 2).

 After INR reversal with prothrombinex and vitamin K, he was taken to the operating theatre. 

Intraoperative findings were of gross dilatation of the sigmoid colon with massive faecal loading. A small rectal perforation approximately 1 cm in diameter was identified in the antimesenteric border. In view of the patient's advanced age, comorbidities, and the absence of intraperitoneal faecal contamination, he was manually disimpacted and the perforation wedge resected then primarily repaired.

 The patient was subsequently admitted to the intensive care unit (ICU) for observation and ventilatory support.

 His postoperative recovery was remarkable for a second perforation picked up with abdominal imaging on day 11 in ICU, after he was thought to have abdominal tenderness. However, since the patient remained nonperitonitic with no fever or signs of deterioration, he was successfully managed conservatively. The patient tolerated diet, was stepped down to the ward, and was subsequently discharged home with regular laxatives. At a follow-up visit one month after discharge, he was back to baseline with normal functioning for himself.

## 3. Case Discussion

Short bowel syndrome (SBS) is a complex disease that results from surgical resection, congenital defect, or disease-associated loss of absorption and is characterized by the inability to maintain protein-energy, fluid, electrolyte, or micronutrient balances when on a normal diet [1]. It is defined in adults as <200 cm of small intestine [2]. SBS presents clinically as chronic diarrhoea and severe wasting.

 SBS is still a relatively rare condition with a prevalence estimated at 2 per million people in Europe. Patients often adapt clinically to the significantly reduced energy absorption associated with SBS through hyperphagia. However, the intestine adapts as well to ensure more efficient absorption per unit length. After massive enterectomy, the intestine hypertrophies and becomes more efficient in nutrient absorption; there is slight lengthening, but more importantly, diameter and villus height increase, effectively increasing the absorptive surface [2].

 The patients with the greatest risk for development of SBS are those with a duodenostomy or jejunoileal anastomosis and <35 cm of residual small intestine, jejunocolic or ileocolic anastomosis, and <60 cm of residual small intestine or end jejunostomy with <115 cm of residual small intestine [3]. Patients with residual colon in continuity will have enhanced energy and fluid absorption and hence can tolerate greater loss of small intestine and retain their nutritional autonomy.

 Without colon in continuity, there is a loss of inhibition on gastric emptying and intestinal transit, which is related to a significant decrease in peptide YY (PYY), glucagon-like peptide I (GLP-I), and neurotensin [4]. PYY is normally released from the L cells in the ileum and colon when stimulated by fat or bile salts. Obviously, these cells are missing in patients who had a distal ileal and colonic resection.

 Stercoral perforation is defined as perforation of the bowel due to pressure necrosis from faecal masses. The latter being no more than an accumulation of stool that has hardened and has remained stationary in the bowel over a long period of time causing stagnation and colonic deformity [5].

 This condition is primarily caused by chronic constipation with at least 61% of patients having a positive history and 100% of patients showing evidence of faecal impaction on the abdominal films [6].

 The majority (77%) of stercoral perforations occur in the sigmoid and the rectosigmoid regions [7].

 This is explained in part by the high rate of absorption of water from the stool in this part of the bowels thus making the water content in the stools the lowest in the distal colon. The narrow lumen of the distal colon also contributes by causing increased intraluminal pressure, which can become higher than the intestinal capillary perfusion pressure, causing necrosis on the antimesenteric border in the event of faecal impaction [8].

 As far as we are aware, this is the first reported case of stercoral perforation in a patient with short bowel.
